# The management of cardiovascular disease in the Netherlands: analysis of different programmes

**DOI:** 10.5334/ijic.889

**Published:** 2013-08-07

**Authors:** Jane M. Cramm, Apostolos Tsiachristas, Bethany H. Walters, Samantha A. Adams, Roland Bal, Robbert Huijsman, Maureen P.M.H. Rutten-Van Mölken, Anna P. Nieboer

**Affiliations:** Institute of Health Policy and Management, Erasmus University, Rotterdam, The Netherlands; Institute of Health Policy and Management, Erasmus University, Rotterdam, The Netherlands; Institute of Health Policy and Management, Erasmus University, Rotterdam, The Netherlands; Institute of Health Policy and Management, Erasmus University, Rotterdam, The Netherlands; Institute of Health Policy and Management, Erasmus University, Rotterdam, The Netherlands; Institute of Health Policy and Management, Erasmus University, Rotterdam, The Netherlands; Institute of Health Policy and Management, Erasmus University, Rotterdam, The Netherlands; Institute of Health Policy and Management, Erasmus University, Rotterdam, The Netherlands

**Keywords:** cardiovascular disease management, integrated care pathways, chronic care delivery, programme implementation, the Netherlands

## Abstract

**Background:**

Disease management programmes are increasingly used to improve the efficacy and effectiveness of chronic care delivery. But, disease management programme development and implementation is a complex undertaking that requires effective decision-making. Choices made in the earliest phases of programme development are crucial, as they ultimately impact costs, outcomes and sustainability.

**Methods:**

To increase our understanding of the choices that primary healthcare practices face when implementing such programmes and to stimulate successful implementation and sustainability, we compared the early implementation of eight cardiovascular disease management programmes initiated and managed by healthcare practices in various regions of the Netherlands. Using a mixed-methods design, we identified differences in and challenges to programme implementation in terms of context, patient characteristics, disease management level, healthcare utilisation costs, development costs and health-related quality of life.

**Results:**

Shifting to a multidisciplinary, patient-centred care pathway approach to disease management is demanding for organisations, professionals and patients, and is especially vulnerable when sustainable change is the goal. Funding is an important barrier to sustainable implementation of cardiovascular disease management programmes, although development costs of the individual programmes varied considerably in relation to the length of the development period. The large number of professionals involved in combination with duration of programme development was the largest cost drivers. While Information and Communication Technology systems to support the new care pathways did not directly contribute to higher costs, delays in implementation indirectly did.

**Conclusions:**

Developing and implementing cardiovascular disease management programmes is time-consuming and challenging. Multidisciplinary, patient-centred care demands multifaceted changes in routine care. As care pathways become more complex, they also become more expensive. Better preparedness and training can prevent unnecessary delays during the implementation period and are crucial to reducing costs.

## Introduction

Chronic diseases are major causes of death and disability worldwide, and the prevalence of such diseases is increasing [1]. They pose a significant health threat and an increasing challenge to healthcare systems. Despite advances in treatment, patients with chronic diseases often do not receive optimal care [2]. Because the causes of chronic diseases are complex, treatment should be multifaceted, integrated, and tailored to patient needs [3].

To improve the efficiency and effectiveness of chronic care delivery, health systems increasingly develop and implement structured disease management programmes as an integrated part of primary care [4–6]. We argue that these programmes can be considered as specific forms of integrated care pathways, focused on providing multi-disciplinary, patient-centred care. Two types of disease management models have been presented in the literature: (1) commercial disease management programmes and (2) primary care disease management programmes based on the chronic care model introduced by Edward Wagner [6]. The chronic care model was developed as a foundation for redesigning primary care practices and improving the quality of chronic care. Whereas commercialised disease management programmes target only patients, those based on the chronic care model are aimed at both patients and professionals, providing an organised multidisciplinary approach to the delivery of care and stimulating communication between professionals and well-informed patients [7]. In the Netherlands, disease management programmes are based on the chronic care model [7–11], albeit adjusted to fit with Dutch healthcare practices [12]. The chronic care model forms the basis for effective chronic care management and addresses shortcomings in acute care models by identifying essential elements that encourage high-quality chronic care delivery [13–15] through the combination of patient-related, professional-directed, and organisational interventions [16,17]. It includes six interrelated components of quality of chronic care delivery: ‘self-management support’, ‘delivery system design’, ‘decision support’, ‘clinical information systems’, ‘healthcare organisation’, and ‘community linkages’ [13–15]. Primary care practices that employ the chronic care model support self-management abilities of chronically ill patients through education, lifestyle programs, and skills building (self-management support), redesign the way care is delivered to chronically ill patients (delivery system design), use evidence (e.g. care standards and clinical guidelines) to provide quality of care (decision support), and implement information systems to improve communication and coordination among professionals, provide timely reminders, feedback, and other methods that increased their visibility at the time of clinical decision-making, monitor effectiveness of care for individual patients (clinical information systems) [7,18]. These four dimensions of chronic care delivery in primary care practices are situated in the larger context of health systems that value and provide incentives for improved quality of chronic care delivery (healthcare organisation) and a community that supports chronic care delivery (community linkages) [18].

Effective disease management is best accomplished by a combination of multiple interventions and collaborations among various professionals, with the support of a variety of health care resources; however, many organisational options are available and programme designers face numerous choices and challenges. Moreover, programmes based on the chronic care model are complex, time-consuming, and costly to implement. Disease management programme implementation therefore requires effective decision making by primary care practices– a process that is increasingly difficult in a time of simultaneous reduced health care budgets and pressure to increase effectiveness and efficiency.

In absence of a ‘one size fits all’ model, practices intending to develop and implement a disease management programme struggle with the multitude of available choices. Even for a single chronic disease, approaches chosen in practice may vary widely, especially in different contexts or settings. This diversity may lead to varying programme costs, health outcomes and improvements in care delivery, depending upon the health care setting, disease, and/or target group [19,20]. Evidence of the effects of disease management programmes on quality of care delivery, quality of life outcomes and efficiency is largely inconclusive [21–23]. Earlier studies, for example, showed that some of the elements of the chronic care model are implemented by care practices with more ease and in greater depth compared to others [24]. Their study revealed that information and communication systems received the most attention, while community linkages received the least attention. Furthermore, in a meta-analysis on cost-effectiveness of the chronic care model in the new millennium Coleman and colleagues [25] concluded that results vary widely and the cost-effectiveness of the chronic care model is just beginning to emerge. In addition, Tsai and colleagues [23] found in their meta-analysis that results on effectiveness of implementing interventions that incorporate one or more elements of the chronic care model for quality of life of patients were mixed. These variations may be explained, in part, by the choices made in the early stages of programme design and implementation.

In this paper, we describe the varieties in patient characteristics, quality of chronic care delivery, health care utilisation costs, development costs, and patient outcomes among newly developed cardiovascular disease management programmes in the Netherlands. We followed eight cardiovascular disease management programmes during the early stages of implementation in various Dutch regions. Given the challenging task of implementing complex, multi-component interventions and of transitioning from acute to (multidisciplinary, patient-centred) chronic care pathways, we examined the processes and challenges of developing and implementing cardiovascular disease management programmes in the Netherlands. We investigated differences among these programmes in terms of context, patient characteristics, disease management level, health care utilisation costs, programme development costs, and patients’ health-related quality of life.

## Methods

### Setting

Our study was performed in the context of a national programme on ‘disease management of chronic diseases’. Requirements of the national programme were that the practices had to have some experience with the delivery of integrated chronic care and were equipped to implement multiple systems needed for the delivery of sufficient chronic care, which resulted in the inclusion of 22 disease management programmes (out of 38 applications to participate in the national programme). These disease management programmes targeted several patient populations: cardiovascular diseases, chronic obstructive pulmonary disease, diabetes, heart failure, stroke, patients with multiple of these morbidities, depression, psychotic diseases, and eating disorders. For this study we selected the eight cardiovascular disease management programmes known as the ‘Vitale Vaten’ project. These eight projects implemented disease management interventions in 39 healthcare practices in eight regions in the Netherlands (see Appendix for an overview of regions and implemented interventions). To describe these eight cardiovascular disease management programmes, we used a concurrent nested mixed-methods approach [26]. We collected baseline quantitative data on the patient and organisational levels during the early implementation stage of the eight programmes. We also conducted baseline interviews with project leaders from all cardiovascular disease management programmes (*n*=8) and additional in-depth interviews with managers and caregivers of one programme (*n*=3) to identify barriers and challenges during early programme implementation. We further describe the research setting, methods and analysis. A detailed description of the methods we employed in our research can also be found in our study protocol [27].

Although the care provision structure varies among the eight programmes, they share target patient groups and most include collaboration between general practitioners, physiotherapists, and dieticians, as well as related practice redesign aimed at improving effective chronic-care management. The disease management programmes aim to overcome shortcomings in acute care models by implementing elements that encourage high-quality chronic disease care.

Each cardiovascular disease management programme consists of a combination of patient-related, professionally directed, and organisational interventions (see Appendix for detailed programme information).

### Patient-related interventions

All eight cardiovascular disease management programmes included self-management interventions, e.g. patient education on lifestyle, regulatory skills, and/or proactive coping.

### Professionally directed interventions

Implementation of the disease management programmes was based on a set of carestandards, guidelines, and protocols and supported by information and communications technology tools such as integrated information systems. All programmes provided training for care providers. There was some variance in profession-specific items in the implementation strategies for professional interventions.

### Organisational interventions

These interventions varied among cardiovascular disease management programmes. Examples include new care provider collaborations, reallocation of tasks, more effective information transfer and appointment scheduling, and case management. Some organisational interventions were related to professional interventions: employing new types of health professionals, redefining professionals’ roles and/or redistributing their tasks, re-structuring interaction between professionals, and planning regular follow-up meetings by the care team.

### Patients

Although all eight disease management programmes focus on patients at risk of (repeated) cardiovascular incident, they targeted different patient populations (Table 1). Two programmes focus exclusively on patients with a history of cardiovascular incidents, three focus exclusively on high-risk patients, one focuses on a combination of patients with previous incidents and high risk patients and the remaining two focus on patients with either high or low risk for cardiovascular disease. No additional inclusion criteria were applied in the programmes. A questionnaire was sent to the 2760 enrolees of the eight cardiovascular disease management programmes to acquire baseline measurements. The response rate was 54% (*n*=1484). The study was approved by the ethics committee of the Erasmus University Medical Centre of Rotterdam in September 2009. Data were collected anonymously and treated confidentially to protect sensitive patient information.

### Quantitative study - measurements

The quantitative study used the following outcome measurements: differences in perceived disease management level, patient characteristics, costs of both programme development and healthcare utilisation, and health-related quality of life. Diseasemanagement level from the patient's perspective was ascertained by administering the 20-item Patient Assessment of Chronic Illness Care (PACIC) questionnaire [28–30]. The Patient Assessment of Chronic Illness Care measures, from the patient's perspective, the extent to which the last 6 months of delivered care aligns with the chronic care model. Its subscales address (1) patient activation, (2) delivery system design, (3) goal setting, (4) problem solving, and (5) follow-up/coordination. Example of items are: when I received care for my chronic illness I was given choices on treatment to think about; satisfied that my care was well organised; asked how my chronic illness affects my life; told how my visits with other types of doctors, like the cardiac surgeon, helped my treatment. The Patient Assessment of Chronic Illness Care was scored by summing each participant's responses to all 20 items, then dividing by 20, the number of items in the scale. Missing values were replaced by mean scale scores if respondents filled in at least 2/3 of the items of a scale. Scores thus ranged from 1 to 5, with higher scores indicating higher quality of chronic care delivery as perceived by patients.

The EuroQol-5 Dimensions questionnaire was used to estimate the utility that patients attached to their health status [31]. The questionnaire consists of five dimensions (mobility, self-care, usual activities, pain/discomfort, anxiety/depression) each of which can take one of three answering categories. Utilities were calculated using the Dutch EuroQol-5 Dimension values set [32]. Basic demographic data on age, gender, marital status, and educational level were gathered. Educational level was dichotomised into ‘low’ and ‘high’, with low representing no or only some primary/secondary education.

We estimated the development costs for each disease management programme by including costs such as labour costs for brainstorming sessions, professional training costs, material costs, capital costs for Information and Communication Technology that occurred in the preparation phase of a programme. The implementation costs that occurred after the start of providing disease management interventions to the patients (e.g. costs of managing aprogramme, the costs of multidisciplinary team meetings, the costs of materials used for patient education, the costs of keeping the Information and Communication Technology operating etc.) are not included in this analysis. The development costs were collected and estimated using a cost-price analytic tool based on the ‘CostIt’ tool developed by the World Health Organisation [33]. This tool was adjusted to allow cost-price calculation in the context of a disease management programme, which facilitated uniform data collection across the eight programmes. The cost-price calculation requires detailed data on capital costs, labour costs, training costs, material costs, maintenance costs of equipment and technology, etc. This information was collected in face-to-face and telephone interviews with programme managers and financial administrators.

The costs of health care utilisation were also estimated. For this measurement, patients were asked to complete a questionnaire abouttheir health care utilisation in the previous 3 months. The questionnaire included detailed questions about visits to general practitioners, visits to medical specialists, paramedical professionals, nurses, emergency departments, medication use, hospitalisation and ambulance use. Self-reported health care utilisation was multiplied with 2010 unit costs that were mainly obtained from the Dutch manual of guideline prices for use in economic evaluation studies [34].

### Quantitative study - analysis

Descriptive analyses were performed to compare patient characteristics, experiences with chronic care, health-related quality of life, development costs and healthcare utilisation costs across disease management programmes. Differences among programmes were established with chi-squared tests and analysis of variance. Multiple regression analysis was performed to investigate relationships among programmes, patient characteristics, and health-related quality of life.

### Qualitative study - interviews

Baseline interviews were conducted in all of the cardiovascular disease management programmes (n=8) within three months of selection for funding through the national programme. The baseline interviews served multiple purposes: to understand the organisation, roles, and responsibilities of the project team; to learn about the goals of the project from the project leader's point of view and to gain an overview of all projects so that one programme could be selected as a case study site for further in-depth qualitative research (see below). Themes that were briefly mentioned during the baseline interviews could then be addressed in subsequent case study interviews.During the phase of research covered in this paper, additional interviews (n=3) were conducted at the case study site. The eleven interviews were held in Dutch or English, ranged from 60 to 90 minutes, and were recorded and transcribed verbatim.

### Qualitative study - case study

As part of the larger evaluation of the 22 disease management programmes, five case study sites were chosen for in-depth research. Interviews with all of the 22 project managers as well as document analysis were used to select five cases for ethnographic ‘thick descriptions’. Criteria for selection were spread over regions, patient groups and different kinds of targeted interventions. One cardiovascular risk disease management programme (Radboud, see Table 1) was chosen as a case study of the cardiovascular disease management programmes. This programme focuses on lifestyle improvement among high-risk patients, such as weight-loss or smoking-cessation. Much emphasis in the development of the programme has been on creating arrangements that support patient-centred care, such as an enhanced electronic patient record. Two general practitioners lead the project together with one nurse project manager, who is responsible for communicating with the caregivers at the sites.

### Qualitative study – analysis

The interviews were inductively coded in two rounds. *In vivo* coding (creating codes using words from the empirical data, without paraphrasing) of all comments was used in the first round to determine a saturation point (no new information) and generate an inductive code list (paraphrasing and categorising). This list was then used to code all the interviews and generate overarching themes.

## Results of quantitative analysis

Analyses of variance showed that age, gender, marital status, educational levels, disease-management level (from the patient's perspective) and health-related quality of life varied significantly among disease management programmes (all *p*<0.001; Table 2). Maarssenbroek (low and high-risk patients) had the youngest population (mean age=59.80, s.d. 9.65) and Radboud (low and high-risk patients) the oldest (mean age=67.82, s.d. 9.90). The majority of Stichting Eerstelijns Samenwerking Achterveld respondents (diagnosed and high-risk patients) were married (89%); other programmes had substantially lower percentages, especially Onze Lieve Vrouwe Gasthuis (diagnosed patients) (56%) and Regionale Organisatie Huisartsen Amsterdam (diagnosed patients) (61%). More patients at Huizen (high-risk patients) and Maarssenbroek had a high education status (71%), as opposed to 60% in the other programmes. Patient-perceived disease-management level was highest in Rijnstate (high-risk patients) and lowest in Regionale Organisatie Huisartsen Amsterdam. Health-related quality of life was highest at Stichting Eerstelijns Samenwerking Achterveld and lowest at Onze Lieve Vrouwe Gasthuis, Regionale Organisatie Huisartsen Amsterdam and Radboud.

Table 3 presents the associations between disease management programmes, patient characteristics, and disease-management level and health-related quality of life as estimated through multiple regression analysis. Results show that health-related quality of life is significantly lower at Onze Lieve Vrouwe Gasthuis, Regionale Organisatie Huisartsen Amsterdam, Radboud and Huizen. Age (β=−0.11; p≤0.001) and being female (β=−0.11; p≤0.001) are negatively associated with health-related quality of life. Being married (β=0.12; p<0.001) and having a higher level of education (β=0.11; p<0.001) are related to a better quality of life, but patient-assessed disease-management level is not related to quality of life.

### Direct costs of disease management programme development

The development costs of the eight disease management programmes are presented in Table 4. Total development costs varied considerably (from €26,807 to €274,783). Two important factors contributing to this variation in costs were the duration of the development phase (longer duration was associated with higher costs) and the number of different professionals involved in programme development. In all disease management programmes, labour costs accounted for more than two-thirds of total costs. Information and communications technology did not contribute substantially to the total development costs.

### Direct costs of health care utilisation

Table 5 presents the descriptive statistics of the 3-month costs of health care utilisation per disease management programme. The mean total health care costs were €350 per patient. There was a wide variation among programmes, with total health care costs ranging from €252 to €628 per patient. The mean health care professional costs were €258 when averaged over all patients and €305 when averaged over the patients who contacted at least one health care professional during the last 3 months (85% of all patients). Mean hospitalisation costs were €855 averaged over all patients and €7399 per patient who had at least one hospital admission (1% of all patients). The mean pharmaceutical costs were €31 per patient and €37 per patient who reported medication use (95% of all patients).

## Results of qualitative analysis

Developing and implementing a patient-centred disease management care pathway with multiple providers requires a transformation for both the providers and the patients. Although research has shown that new systems of care for chronic illness can improve delivery of care and patient outcomes [35], their distribution of care responsibilities, and especially the time commitment needed to organise doctors and coordinate with other providers, are prevalent concerns. Project leader interviews revealed initial concerns about organisational challenges, identifying the target population activating patients and proper Information and Communication Technology support for these processes.

### Organisational challenges

In the development and implementation of the disease management programme, the project leaders and manager focused on the organisational challenges of working with large teams and developing a solid basis for programme implementation.

It takes considerable time to clearly get what everyone wants and what everyone already does. I do not know your experience with doctors, but here it is true that every doctor has a different approach and style. There is very little consistency. (Project leader at A)

We could have just started and waited to see what we would encounter. But we wanted to start by laying a good foundation and then trying to build from there. (Interview with H, programme manager)

Although the respondents emphasise laying a ‘good foundation’ and creating consistency in practice, they still encounter challenges in coordinating care between multiple providers. These challenges are magnified as the care pathway extends beyond the General Practitioner office building.

Practices are very large organisations. So first, you must convince people of the importance of doing the research. That takes a lot of effort.... Plus, you should also involve other members of the care teams, the physical therapist, the nutritionist. Later, I found a missed opportunity, in that we have no contact with pharmacists. That we just forgot. So, this is probably something we'll have to fix. (Interview with M, X, H)

Communication between the project leadership team and other care providers can be difficult because practices have their own processes, protocols, and priorities in care giving. While the project leadership team placed emphasis on creating a good foundation early in the project timeline, the effort of organising the disease management programme remains a work in progress.

### Activating patients

Through patient-centred care, patients are made responsible for choosing how they want to change (and presumably improve) their lifestyle. The clinician, formerly a directive force in health care, takes on a more collaborative role and decides together with the patient how to manage a given health issue.

We are not used to patient-centred working. The doctor says to the patient, you must stop smoking and eat more healthy food. Then the patient goes home. And yeah, the doctor can tell this, but we let the patients choose for themselves and we hope that we have convinced them. But the patients can choose their own risk factor to change. (Interview with X)

Such developments demonstrate a more general shift in health care towards a more'active’ role for patients, but this approach implies that patients not only have both the knowledge of cardiovascular risk factors and a desire to change behaviours related to these risk factors, but also the ability to make the suggested changes to improve their health and health care.

### Electronic health records

To organise multidisciplinary, patient-centred care pathways, the organisations are implementing electronic health records that connect the various clinicians and allow them to access patient information from any internet-friendly location. But implementing Information and Communication Technology systems brings a new set of challenges:

We have had a lot of barriers. The first barrier is with the ICT system. We didn't receive it already finished… It has been a long road to get to where we are now. (Interview with X) It is not easy – GPs are always very busy. And when the projectstarted, I had to change the daily practices, the rules. They must use another screen and work with patients in a different way than they are used to. So it's not easy to implement this in our daily practices. (Interview with X)

As these quotes show, implementing computer-based systems involves a number of organisational, cultural, and technical changesthat slow progression in the disease management programmes.

## Discussion

In absence of adequate descriptions of on-going cardiovascular disease management programmes,this study described the varieties in patient characteristics, quality of chronic care delivery, health care utilisation costs, development costs, and patient outcomes among newly developed cardiovascular disease management programmes in the Netherlands. Improvements from disease management programmes in terms of care and costs have been documented [19,20], but results vary widely across health care settings and target groups [23–25]. Given the challenging tasks of developing complex, multi-component interventions and transitioning from acute to chronic care, we therefore investigated challenges that occurred during the implementation phase and assessed financial and organisational development costs.

The impacts of cardiovascular disease management programmes are expected to depend on patient characteristics [8,36]. Results of this study showed that patient characteristics, disease-management level, and health-related quality of lifeall varied widely among the cardiovascular disease management programmes. In this cross sectional study, no relationship was found between patients’ perceived disease management level and health-related quality of life. This, however, is not surprising because we studied the early stages of cardiovascular disease management programmes. We expect to find a significant relationship in the long run. Programmes coping with older, less educated patients with poor health-related quality of life potentially have a harder time attaining improved patient outcomes, because it is more difficult for such patients to change lifestyle, adhere to treatment recommendations and ask for additional support. However, if the care being delivered is tailored to the specific needs of these vulnerable groups, it also offers the chance for greater improvement.

Our cost analysis and the description of actions performed within the context of cardiovascular disease management programmes revealed significant differences, with total development costs varying considerably between the eight programmes. The duration of the development period and the number of different disciplines involved in this phase might explain this variation. For example Stichting Gezondheidscentra Eindhoven had the highest development costs due to the relatively long development period (18 months) and the relatively high involvement (in fte's) of many of different disciplines (11) in the preparation of this program. This relatively intensive and lengthy preparation process may reflect the variety and complexity of disease management interventions provided by Stichting Gezondheidscentra Eindhoven.. In general terms, spending much time in the developing phase leads to higher costs due to high labour costs. Although labour costs appear to vary substantially among programmes, they account for more than 80% of total costs in all programmes. Using only baseline data, it is currently not possible to provide further insight into the relationship between the complexity of the programme and the costs. This data is, however, currently being gathered and can be reported on in a later phase.

Our qualitative analysis suggests the need to consider more carefully the potential consequences of developing and implementing complex, multi-component interventions and patient-centred integrated care pathways for primary care. While the primary care setting is thought to be an ideal location for the coordination of care [37], this can be time consuming and significantly costly for practices where coordinated care is a new endeavour, and especially when the goal is sustainable change. While project leaders take steps to be efficient when developing and implementing programmes, as it can be seen in the qualitative data on creating a good foundation, organising and training health care providers is also time-consuming. Although this is largely unavoidable due to the complexities involved in changing practice structures, working with multiple care providers, transforming the patient/provider relationship, and developing computer-based health systems, the resulting delays are important findings since the length of development is the main cost driver.

Given recent attention for high costs of implementing Information and Communication Technology systems in health care [38], we expected information and communications technology costs to be an important cost driver; however, cost analysis results indicated that, during the development phase, they were by far outweighed by the labour costs. Importantly, though, our qualitative study revealed that they could be indirectly significant by delaying the implementation process, thereby increasing the length of the development period, which is the main cost driver. Even when implemented, such systems remain a work in progress and may not provide the improvement in care desired [39,40]. Because the task of system development is usually contracted out to computer programmers and software developers, this reduces the autonomy that project leaders have over the timeline, the computer-based programme, and, consequently, the costs. The important lesson for those wanting to implement cardiovascular disease management programmes is to be prepared for and aware of these challenges.

Some study parameters are notable. Although we evaluated a diverse set of eight cardiovascular disease management programmes, general measures given to each site did yield a solid evidence base. Moreover, in combination with the qualitative descriptions, the variation between sites contributed to deeper understanding of the relationship between the complexity of a project, implementation challenges and related costs. The response rate of 54% among patients may have affected our study findings. For example if non-responders mainly consisted of patients with more severe conditions health-related quality of life may be overestimated. Furthermore, response rates among practices with lower socioeconomic status patients were lower, which indicates selection bias and possible confounding.

We do not know to what extent other policy issues influenced the development and implementation of the disease management programmes. For example the Dutch Ministry of Health has recently introduced an integrated payment system, the Chain-Diagnosis Treatment Combination (chain-DTC), which combines the costs of multiple professionals working primarily in general or primary care and, to a limited extent, in specialised or hospital-based outpatient care. The chain-DTC stimulates cooperation among different providers of curative interventions in primary-care settings (e.g. General Practitioners, practice assistants, physiotherapists, dieticians). While, expectations of such an integrated payment system were high, practice shows that most cardiovascular disease management programmes are not financed via a chain-DTC yet. The cross-sectional study design meant we could describe only the current situation and relationships. We could not answer the question why some cardiovascular disease management programmes seem to work better than others. Future research will enable us to identify predictors of health-related quality of life and give insight into which types of programme interventions enhance health-related quality of life in cardiovascular disease patient groups. Future research is also necessary to investigate whether a higher disease-management level indeed positively affects health-related quality of life.Becauseour study was conducted in the specific healthcare setting of the Netherlands, comparing results to other countries using the same mixed-methods approach and in-depth description of interventions would be usefulto further our understanding of the influence of different financial structures and cultural settings on creating sustainable integrated care pathways.

## Conclusions

While improvements from disease management programmes in quality of care and cost-effectiveness have been documented in the Netherlands [41–44],the results vary widely across health care settings, diseases, and target groups [23–25]. The current study described eight cardiovascular disease management programmes to give insight into the forms of disease management and the feasibility of a disease management approach. The results showed that disease management level, costs, health care utilisation, patient characteristics, and health-related quality of life of patients all varied widely among the cardiovascular disease management programmes. These variations are expected to influence future programme outcomes, cost-effectiveness and sustainability.

Implementing cardiovascular disease management programmes is time-consuming and challenging because they demand complex, multifaceted changes in routine care. Furthermore, as these care pathways become more complex, they also become more expensive. In the case of cardiovascular disease management programs, costs are to a large extent attributable to delays in implementation.Therefore, better preparedness, incremental implementation plans, and training might reduce the implementation period and, thereby, costs.

## Figures and Tables

**Table 1.**
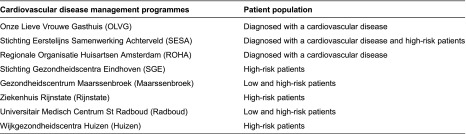
Overview of the cardiovascular disease management programmes

**Table 2.**
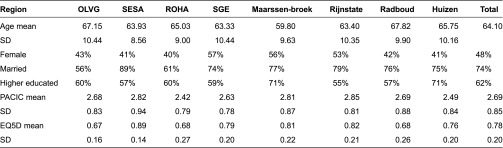
Descriptive statistics of patient characteristics, experiences with chronic care and health-related quality of life

Note: Analysis of variance: Age (*F*_group_ =14.9; *p*<0.001); Female (Chi-square 29.1; *p*<0.001); Married (Chi-square 57.3; *p*<0.001); Higher educated (Chi-square 25.5; *p*<0.001); Pacic (*F*_group_=6.1; *p*<0.001); EQ5D (*F*_group_=6.5; *p*<0.001).

**Table 3.**
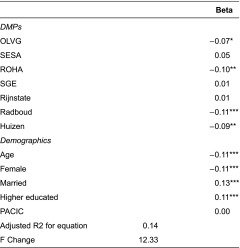
Multiple regression analysis of disease management programmes and patient characteristics on health-related quality of life

Note: Reference care group is Maarssenbroek. DMPs=Disease Management Programmes.

*p≤.05; **p ≤0.01; *** *p* ≤0.001.

**Table 4.**
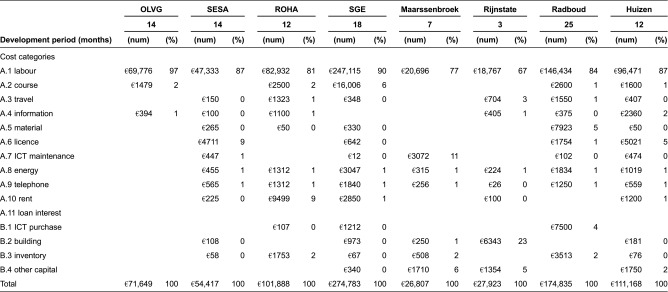
Development costs of eight cardiovascular disease management programmes

**Table 5.**
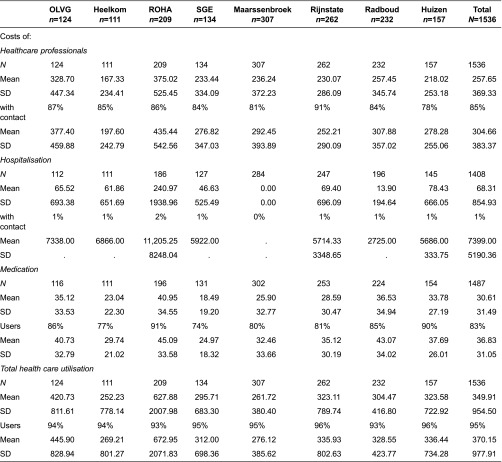
Descriptive statistics of health care utilisation costs in three months period (in euros)

Note: Analysis of variance for health care utilisation. All: care givers (*F*_group_ =5.4; *p*<0.001); hospitalisation (*F*_group_=1.5; *p*>0.05); medication (*F*_group_=9.9; *p*<0.001); total health care utilisation (*F*_group_ =3.4; *p*<0.001); with contact/users: care givers (*F*_group_ =5.6; *p*<0.001); hospitalisation (*F*_group_=0.4; *p*>0.05); medication (*F*_group_=6.8; *p*<0.001); total health care utilisation (*F*_group_ =3.5; *p*<0.001);

## Appendix: overview of the eight cardiovascular disease management programmes

### Onze Lieve Vrouwe Gasthuis (OLVG)

The programme targets cardiovascular disease patients diagnosed with: myocardial infarction; Angina Pectoris; Stroke; Transient Ischaemic Attack; peripheral arterial disease; aortic aneurysm. The core disease management team consists of 6 professionals: the project leader (internist/vascular specialist), the project coordinator (nurse practitioner), two General Practitioners experienced in cardiovascular diseases, a internist/vascular specialist and a student/researcher. The overall disease management team consists of thirty-seven professionals (17 General Practitioners, 4 internists/vascular specialists, 3 cardiologists**, 1 psychiatrist, 1 neurologist, 1 vascular surgeon, 1 nurse practitioner, 8 practice nurses and the manager/project leader) and the cardiovascular disease management programme is implemented in five general practices. This cardiovascular disease management programme focuses on secondary prevention (body weight/blood pressure/cholesterol/smoking/adherence to therapy). Neurologists, vascular surgeons and cardiologists refer patients from the secondary to primary cardiovascular disease management programme, in which they aim to provide care according to the same clinical guidelines used in the hospital.

Patient-directed interventions:

- Use of Electronic Patient Record system that facilitates online communication between patient and provider to enhance self-management.

Professional-directed intervention:

- Implementation of care standard for cardiovascular risk management
- Uniform treatment protocols in primary and hospital care
- Increase knowledge and independence of primary practice nurses and General Practitioners
- Benchmarking and auditing of patient satisfaction

*Organisational intervention*:

- Delegation of care from in-hospital (internist/vascular specialist and specialised nurse practitioner) to General Practitioner and primary care practice nurse
- Redistribution of hospital to primary care
- Regular follow-up
- Intensification of cooperation with physiotherapists in using existing exercise programmes
- Regional transmural Electronic Patient Record system with a patient portal containing information on projects and referral options

### Stichting Eerstelijns Samenwerking Achterveld (SESA)

The programme targets patients diagnosed with cardiovascular disease or high-risk patients. They are located in a rural area with a population that consists of relatively more people younger than 21 years, people above 65 years old, and mainly native Dutch people. They especially focus on promoting a culture change in their patients. In general their patients are not very assertive or actively involved in managing their chronic condition. The disease management team consists of nine professionals (3 General Practitioners, a dietician, a physiotherapist, an administrator, project leader, physician-assistant and a practice nurse) and the cardiovascular disease management programme is implemented in one general practice. They aim to work according to the cardiovascular care standard.

*Patient-directed interventions*:

- Training of patient groups in active participation and self-management (healthy diet, exercise, stress management)

*Professional-directed intervention*:

- Educational meetings on the disease management programme
- Education and training in patient stimulation and support to enhance their active involvement
- Setting quality parameters for auditing and feedback

*Organisational intervention*:

- Delegation of care from General Practitioner to practice nurse, from heart specialist to General Practitioner
- Regular follow-up
- Integrated information system
- Implementation of regional transmural Electronic Patient Record system in 3 years

### Regionale Organisatie Huisartsen Amsterdam (ROHA)

The programme targets patients with a history of a cardiovascular event. The core team consists of seven professionals (4 General Practitioners, project advisor, cardiologist and a physician-assistant cardiovascular diseases) and the cardiovascular disease management programme is implemented in eight general practices. Two General Practitioner practices located in the same region proving care as usual serve as a control practice. Since providing cardiovascular care is relatively new in primary care a project team is set up with professionals working in primary and secondary care. Regular meetings of this project team are expected to create discussion, learning experiences and knowledge.

*Patient-directed interventions*:

- Informational meetings (also in Turkish and Moroccan)
- Community-based lifestyle interventions
- Individual care plans
- Support of self-management with internet, email, text messages, or incentives
- Lifestyle and exercise programmes

*Professional-directed intervention*:

- Basing General Practitioner primary care protocols on the care standard cardiovascular risk management and the Dutch College of General Practitioners (NHG) guidelines
- Education and training for lifestyle programmes
- Training cycle for General Practitioners
- Education of practice nurses in cardiovascular disease care
- Use of the Information and Communication Technology*** system for benchmarking

*Organisational intervention*:

- Use of the patient platform to implement self-management programmes
- Communication with local immigrant organisations to identify and mitigate potential barriers to immigrants’ involvement in disease management programmes
- Redistribution of hospital to primary care
- Delegation of care from specialist to General Practitioner or practice nurse
- Regular follow-up
- Transmural care chain
- Cooperative agreements between primary and hospital care
- Special policies for immigrants
- Investigation of (im)possibilities for chain-integrated information system with a patient portal

### Stichting Gezondheidscentra Eindhoven (SGE)

The programme targets high risk cardiovascular disease patients. The disease management team consists of one hundred professionals (46 General Practitioners, 27 physiotherapists, 17 practice nurses, 10 dieticians) and the cardiovascular disease management programme is implemented in ten general practices. Care for cardiovascular disease patients is already implemented within the General Practitioner practices, but needs structure and coordination.

*Patient-directed interventions*:

- Patient education
- Personal coaching
- Motivational interviewing
- Facilitation of self-monitoring and self-management
- Customised programmes to quit smoking, exercise, maintain healthy diet and develop coping skills

*Professional-directed intervention*:

- Disease management programme education
- Training in motivational training
- Use of validated performance and process indicators as quality parameters for auditing and feedback
- Individual monitoring of patients and evaluating quality of care at group level
- Provision of feedback and suggestions for improvement by the care registration team

*Organisational intervention*:

- General Practitioner as the central care provider in close collaboration with the practice nurse
- Emphasis of care providers’ coaching role
- Exercise programmes provided by a consultant at regular sports facilities
- Regular follow-up
- Cooperation with diagnostic centres and hospital care specialists
- Cooperative agreements between primary and hospital care
- Early detection of high-risk patients
- Uniform systematic registration by all professionals
- Support of ICT-registration system for monitoring and feedback

### Gezondheidscentrum Maarssenbroek

The programme targets patients at risk for cardiovascular disease. Low risk patients are seen annually and high risk patients will receive lifestyle interventions. Patients with a history of cardiovascular diseases stay within secondary care if necessary. The disease management team consists of ten professionals (2 General Practitioners, internist, project leader, a nurse practitioner, physiotherapist and four practice nurses) and the cardiovascular disease management programme is implemented in one general practice. They had already implemented some elements of cardiovascular care but with the disease management programme they aim to expand cardiovascular care in a primary setting.

*Patient-directed interventions*:

- Lifestyle advisors and plans (exercise, diet, quit smoking)
- Exercise programmes
- Quit smoking consultation hours
- Development of individual patient care plans based on their risk profiles
- Motivational interviewing

*Professional-directed intervention*:

- Cardiovascular programme based on the care standard for cardiovascular risk management
- Training in motivational interviewing
- Setting quality parameters for auditing and feedback
- Benchmarking
- Regular intervention and goals evaluation by central care director

*Organisational intervention*:

- Mapping of patients’ wishes and active involvement of patients or patient groups in the cardiovascular disease programme
- Annual patient satisfaction inquiry
- Central care director (nurse practitioner) responsible for content of the care plan, delegation of lifestyle interventions to the lifestyle advisor, regular meetings with the pharmacist on patients’ medication use, and proactive contact with other involved professionals
- Collaboration of lifestyle advisor and patient on coaching and lifestyle plans
- Cardiovascular disease practice nurse consultation hours 4 times a week. Patient inflow through General Practitioner and active involvement with at-risk patients
- Shared decision making and actively reminding patients of their decisions and treatment plans
- Regular follow-up
- Expansion of chain care to hospital care
- Transmural protocol
- Chain-integrated information system with a patient portal

### Ziekenhuis Rijnstate

The programme targets high-risk cardiovascular disease patients. The core of the disease management team consists of six of professionals (General Practitioner, internist, cardiovascular nurse, project leader and 2 practice nurses) and the cardiovascular disease management programme is implemented in ten general practices. The innovative element of this cardiovascular disease management programme is its transmural nature. They aim to transfer knowledge and expertise in cardiovascular care from secondary to primary care.

*Patient-directed interventions*:

- Joint medical consultations for primary care and hospital care patients (n=10), a spouse/family member, and a physician

*Professional-directed intervention*:

- Improved implementation of guidelines for cardiovascular care in primary and hospital settings
- Implementation of care standard for cardiovascular risk management to improve implementation of the Dutch College of General Practitioners (NHG) guidelines in primary and hospital care settings
- Education for General Practitioners
- Cooperative learning among nurses at the outpatient clinic

*Organisational intervention*:

- Knowledge exchange
- One-stop outpatient clinic
- Joint consultation hours
- Regular follow-up in primary care
- Cooperation between primary and hospital care
- Uniform treatment plan in primary and hospital care
- Early recognition of high-risk patients at outpatient clinics and general practices
- Transmural Electronic Patient Record system vascular risk management with a patient portal
- Development of transmural chain Diagnosis Treatment Combination

### Universitair Medisch Centrum St Radboud

The programme targets patient with the following criteria:

1. At least a year under treatment of a General Practitioner or specialist due to cardiovascular disease risk.
2. Primarily starting with secondary prevention.

They also focus on patients with low socioeconomic backgrounds. The disease management team consists of twenty-three professionals (11 General Practitioners, 3 physiotherapists, 2 dieticians, a internist, project leader and 5 practice nurses) and the cardiovascular disease management programme is implemented in two general practices and two general practices in the area providing usual care serve as control group. They aim to establish systematic transmural collaborative care in the disease management programme for which they will implement a structure that enhances cooperation between professionals within the disease management programme.

*Patient-directed interventions*:

- Patient-driven choice programme: patient can choose a central care provider, the risk factor(s) he/she wants to tackle, the intervention(s), personal goals, and use of web-based support
- Individual care plans with personal goals
- Motivational interviewing
- Self-management support with vulnerable groups
- Cognitive behavioural therapy

*Professional-directed intervention*:

- Implementation of care standard for cardiovascular risk management
- Training in motivational interviewing
- Education of professionals
- Auditing and feedback sessions to improve quality of patient satisfaction and outcomes

*Organisational intervention*:

- Internist and vascular nurse as central care providers
- Regular follow-up
- Contact with unmotivated patients every 3 months
- Development of transmural collaborative care structure
- Enhanced interaction and cooperation among professionals involved in the disease management programme on referrals and treatment plans
- Contact with patients of low socioeconomic status and different cultural backgrounds
- Development of online patient files accessible to all professionals and the patient
- Registration of risk profiles in the ICT system

### Wijkgezondheidscentra Huizen

The programme targets patients with a higher risk for cardiovascular diseases. The disease management team consists of eight professionals (2 General Practitioners, practice nurse, project leader, pharmacist, physiotherapist, dietician and manager) and the cardiovascular disease management programme is implemented in two general practices.

*Patient-directed interventions*:

- Education of patients to enhance self-management skills
- Individual care plans
- Motivational interviewing
- Exercise programmes
- Quit smoking counselling
- Healthy diet counselling
- Web-based support programmes to enhance self-management (access patient file, information, e-consultation)

*Professional-directed intervention*:

- Implementation of care standard for cardiovascular risk management
- Education of professionals on informing patients and training in motivational interviewing
- Education of physician's assistants on cardiovascular risk factors
- Auditing and feedback on performance indicators and benchmarking

*Organisational intervention*:

- Consultation with several patient groups
- Patient satisfaction research
- Delegation of care from General Practitioner to practice nurse
- Practice nurse as central care provider
- Identification of patient groups based on risk profiles
- Pharmacist monitoring of medication
- Physical therapist-run exercise programmes
- Healthy diet counselling from practice nurse in collaboration with dietician
- Development of multidisciplinary treatment programme for obesity
- Registration of risk profiles
- Structural knowledge exchange
- Regular follow-up
- Cardiovascular counselling hours
- Cooperation between primary and hospital care
- Development of a multidisciplinary programme for obesity
- Professional information exchange in the ICT system
